# Bioinformatics Analysis Reveals an Association between Autophagy, Prognosis, Tumor Microenvironment, and Immunotherapy in Osteosarcoma

**DOI:** 10.1155/2022/4220331

**Published:** 2022-07-14

**Authors:** Yibo Ma, Changgui Tong, Mingjun Xu, Hongtao He, Changjian Chen

**Affiliations:** ^1^Graduate School of Dalian Medical University, Dalian Medical University, Dalian 116044, China; ^2^The Fifth Ward of Department of Orthopedics, The Second Hospital of Dalian Medical University, Dalian 116000, China; ^3^The Second Hospital of Dalian Medical University, Dalian Medical University, Dalian 116000, China; ^4^The Third Ward of Department of Orthopedics, The Second Hospital of Dalian Medical University, Dalian 116000, China; ^5^The First Ward of Department of Orthopedics, The Second Hospital of Dalian Medical University, Dalian 116000, China

## Abstract

Autophagy is a catabolic pathway involved in the regulation of bone homeostasis. We explore clinical correlation of autophagy-related key molecules to establish risk signature for predicting the prognosis, tumor microenvironment (TME), and immunotherapy response of osteosarcoma. Single cell RNA sequencing data from GSE162454 dataset distinguished malignant cells from normal cells in osteosarcoma. Autophagy-related genes (ARGs) were extracted from the established risk signature of the Molecular Signatures Database of Gene Set Enrichment Analysis (GSEA) by univariate Cox and least absolute shrinkage and selection operator (LASSO) Cox regression analysis. Overall survival (OS), TME score, abundance of infiltrating immune cells, and response to immune-checkpoint blockade (ICB) treatment in patients with different risks were compared based on risk score. Nine ARGs were identified and risk signature was constructed. In all osteosarcoma datasets, the OS was significantly longer in the high-risk patients than low-risk onset. Risk signature significantly stratified clinical outcomes, including OS, metastatic status, and survival status. Risk signature was an independent variable for predicting osteosarcoma OS and showed high accuracy. A nomogram based on risk signature and metastases was developed. The calibration curve confirmed the consistency in 1-year, 3-year, and 5-year predicted OS and the actual OS. The risk score was related to 6 kinds of T cells and macrophages, myeloid-derived suppressor cell, natural killer cell, immune score, and stromal score in TME. The risk signature helped in predicting patients' response to anti-PD1/anti-PD-L1 treatment. The ARGs-led risk signature has important value for survival prediction, risk stratification, tumor microenvironment, and immune response evaluation of osteosarcoma.

## 1. Introduction

Osteosarcoma is the main malignant primary bone tumor in young adults and children. Osteosarcomas are derived from osteoblast mesenchymal stem cells (MCSs) and are most common in the metaphysis of the long bone, especially around the knee joint at the distal femur or proximal tibia [1]. Three main histological subtypes of osteosarcoma have been defined: osteoblastic osteosarcoma (50% of cases), chondroblastic osteosarcoma (25% of cases), and fibroblastic osteosarcoma (25% of cases) [2]. With complete surgical resection and multiagent chemotherapy, up to 70% of patients with high-grade osteosarcomas and localized extremity tumors can become long-term survivors [3]. However, for patients with metastatic diseases or patients with recurrence after treatment, the survival rate after five years was less than 30% [2]. The high level of cellular heterogeneity and the complexity of molecular and genetic mechanisms associated with osteosarcoma make the clinical treatment of metastatic osteosarcoma extremely difficult [4]. For the past few years, the increasing feasibility of molecular profiling together with the creation of both robust model systems [5–7] and large, well-annotated tissue banks has led to an increased understanding of osteosarcoma biology [8].

Autophagy is an intracellular degradation process that participates in the regulation of osteoblast homeostasis, including the survival, differentiation, and activity of osteoblasts, osteocytes, and osteoclasts [9]. A series of evidence suggests that autophagy defects may be related to some bone diseases, such as osteoporosis [10] and Paget's disease of bone [11]. It is reported that autophagy is a survival-promoting way for tumor cells to increase their proliferation and development, resist cancer therapy, and retain cancer stem cell (CSC) pools in osteosarcoma [12]. Moreover, the molecular network of autophagy can control the chemical resistance of osteosarcoma in tumor microenvironment (TME) and mechanism [13]. Therefore, targeted autophagy is a promising therapeutic strategy to overcome chemotherapy resistance and reduce metastasis of osteosarcoma [14]. At present, the research on autophagy in osteosarcoma only stays at the molecular level, involving complex TME and a variety of molecular mechanisms cannot be ignored.

In this study, we focused on the comprehensive analysis of scRNA-seq and RNA-seq to explore the potential effects of autophagy-related molecules on the prognosis, TME, and immunotherapy of osteosarcoma, which provided a potential direction for the study of extensive microenvironmental effects and regulatory mechanisms of autophagy regulatory molecules in osteosarcoma.

## 2. Materials and Methods

### 2.1. Collection and Analysis of ScRNA-Seq Data

The expression profiles and clinical data of 50174 cells from 6 osteosarcoma samples were downloaded from the GSE162454 dataset in the Gene Expression Omnibus (GEO, https://www.ncbi.nlm.nih.gov/geo/) database. Seurat in *R* software was used to process scRNA data, and the proportion of mitochondria and rRNA was calculated by PercentageFeatureSet function to ensure that the motifs expressed in each cell were more than 100 and less than 8000, mitochondrial gene content <35% and unique molecular identifiers (UMI) > 1000. ScRNA-seq data were normalized via the “LogNormalize” algorithm. The highly variable genes were identified by FindVariableGenes function and principal component analysis (PCA) was performed based on the first 2000 highly variable genes. The first 50 PCs were selected to use *t*-distributed stochastic neighbor embedding (*t*-SNE) algorithm for dimensionality reduction. The CellCycleScoring function was applied to identify the cycle phase-specific changes of cells in different samples. The “CellCycleScoring” function scores each cell according to the expression of *G*2/*M* and *S* phase markers. The *G*2/*M* or *S* phase scores were inversely correlated, and the cells that did not express *G*2/*M* and *S* phase markers were in *G*1 phase [15].

### 2.2. Clinical Data Download and Processing of Osteosarcoma Samples in Target Database

Clinical phenotypic data and expression profiles of osteosarcoma samples were downloaded from the Target database. Samples lacking survival time and survival status were eliminated, and survival time of all patients >0 years were ensured. Finally, 84 osteosarcoma samples with complete clinical data were included. In addition, 53, 47, and 34 tumor samples with complete clinical data were downloaded from the three GEO datasets GSE21257, GSE39058, and GSE16091, respectively.

### 2.3. Gene Set Variation Analysis (GSVA)

The Hallmark gene set was obtained from Molecular Signatures Database (MsigDB, https://www.gsea-msigdb.org/gsea/msigdb/). 531 autophagy‐related genes (ARGs) were extracted from the MsigDB of GSEA, https://www.gsea-msigdb.org/gsea/index.jsp). “GSVA” performed ssGSEA on normal and tumor cells from tumor samples in Target database to determine enrichment pathways.

### 2.4. Construction of Risk Signature Based on Autophagy-Related Genes

To screen the genes related to prognosis from the obtained ARGs, univariate Cox regression analysis was performed. Then, LASSO regression was performed with 10-fold cross validation and a *p* value of 0.05 was based on the results of the univariate analysis for ARGs. Then stepwise multivariate Cox regression analysis was performed, and the filtered ARGs were used to build a risk signature. According to the risk signature, the risk score of each patient in training and verification set was obtained, and the standardized risk score was divided into high-score and low-score groups with a critical value of 0.

### 2.5. Prognostic Value Evaluation of Risk Signature

The *R* software package “timeROC” determined the AUC of the model at different time points by generating the time-dependent receiver operating characteristic (ROC) curve of risk signature. Kaplan-Meier survival analysis was carried out to distinguish the OS of high-score group and low-score group. The *T*-test was applied to explore the correlations between the risk score and clinicopathological features (including initially diagnosed age, gender, metastasis, and life status).

### 2.6. Construction of a Nomogram

Univariate and multivariate Cox regression analyses were performed to determine the independent prognostic factors of osteosarcoma. Based on all the independent prognostic factors screened, nomogram was drawn using “rms.” The predictive performance of nomogram was evaluated by generating 1-year, 3-year, and 5-year calibration chart and comparing them with the benefit rate of a single index by decision curve analysis (DCA).

### 2.7. The Tumor-Infiltrating Immune Cells and TME Score of Risk Signature

To determine the relationship between risk signature and tumor-infiltrating immune cells, the proportion of infiltrating immune cells was calculated by CIBERSORT and compared between high- and low-risk score groups. The TME score of each sample, including stromal score, immune score, and ESTIMATE score, was calculated by the “estimate” package in *R* based on the gene expression of the samples in the Target dataset.

### 2.8. Acquisition of Immune-Checkpoint Blockade Genomic

To establish the relationship between risk score and ICB, we collected two immunotherapy groups: an anti-PD-L1 antibody from the “IMvigor210” *R* package and an anti-PD-1 antibody from the GSE78220. The gene expression profiles of pretreatment biopsy samples were transformed into TPM format.

### 2.9. Statistical Analysis

A two-tailed unpaired Student's *t*-test was used to compare differences between two the groups of normally distributed data. The correlation between infiltrating immune cells and risk score was calculated by Spearman correlation analysis. Kaplan-Meier statistics and log-rank tests were used for survival analysis. All data processing was conducted in *R* 4.0.1 software and a two-sided *p*value <0.05 was considered significant.

## 3. Results

### 3.1. Identification of Malignant Cells and Normal Cells and Cell Types in Osteosarcoma Using ScRNA-Seq Data

**Figure 1:**
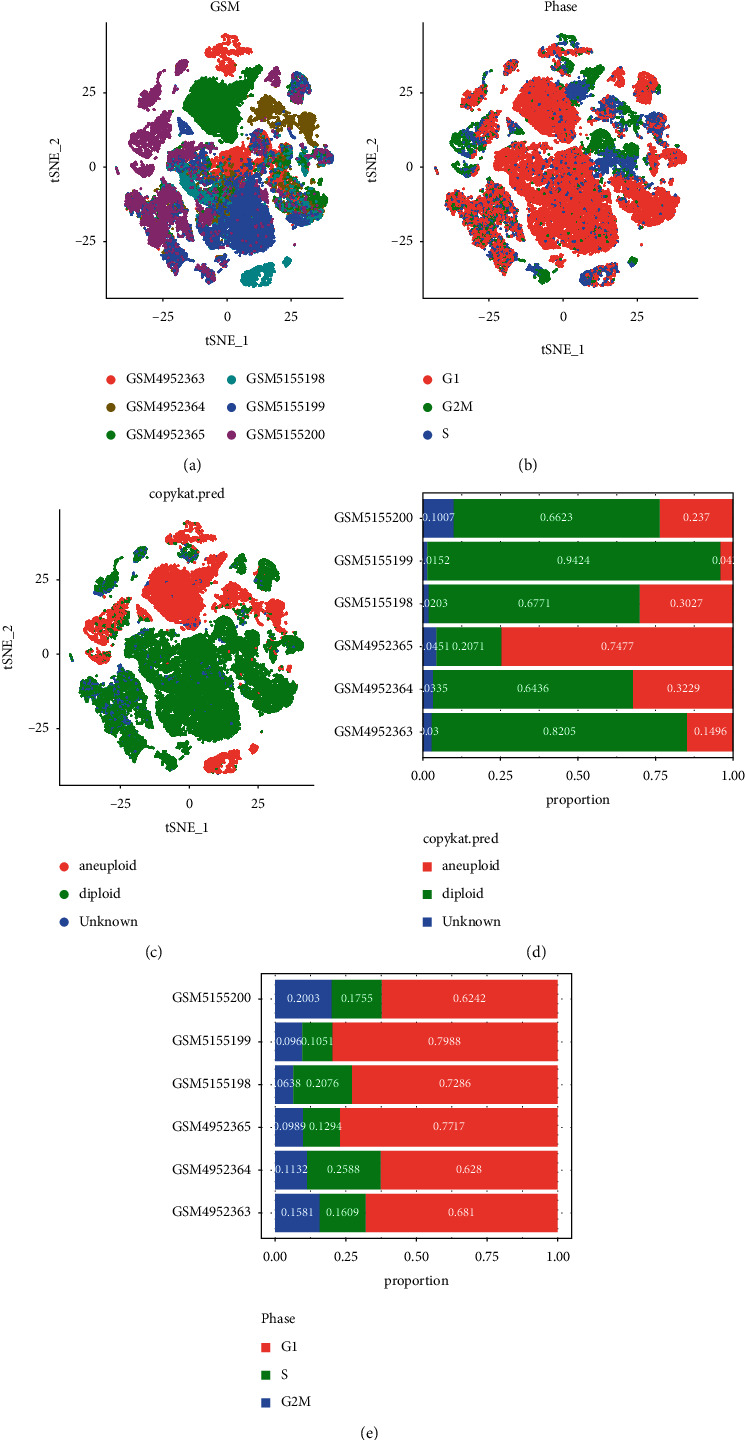
Normal and malignant cells in osteosarcoma (a) A *t*-SNE map of the distribution of cells in each osteosarcoma sample, and each color represents the cells in each sample. (b) The *t*-SNE diagram shows cells at different stages of the cell cycle, and different stages of the cell cycle are marked with different colors. (c) The t-SNE diagrams of malignant and nonmalignant cells in osteosarcoma samples are represented by different colors. (d) The proportion of malignant and nonmalignant cells in each osteosarcoma sample. (e) The proportion of G1, G2/M, and S phase cells in each osteosarcoma sample.

**Figure 2:**
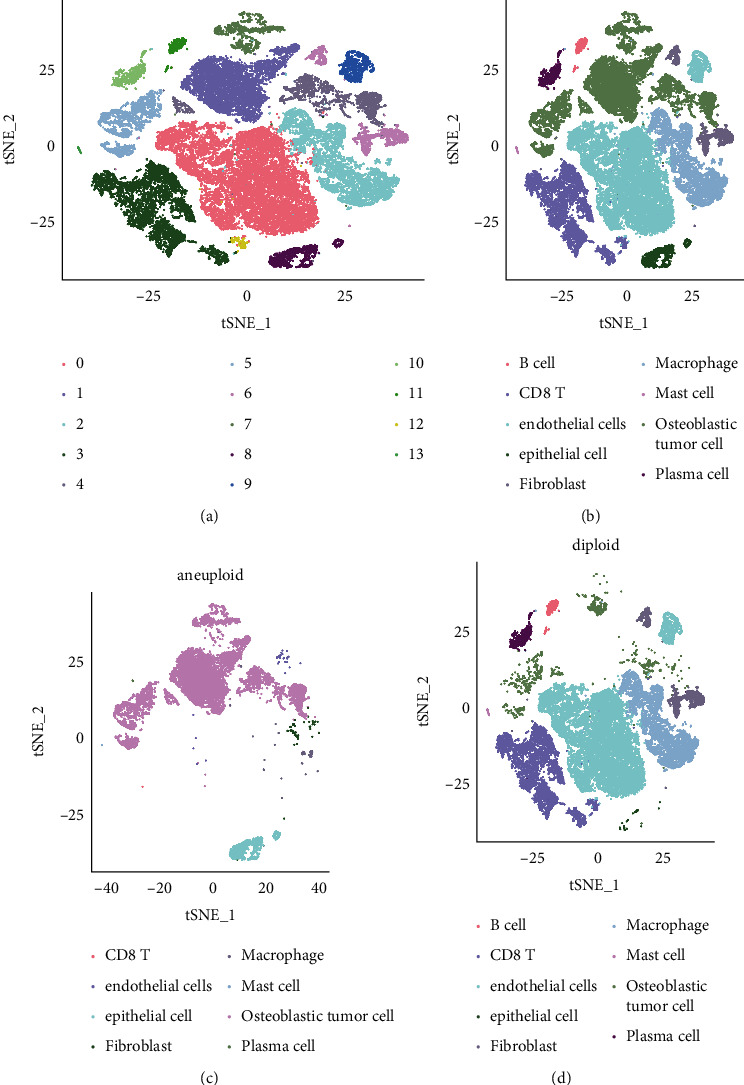
The cell types contained in malignant and nonmalignant cells. (a) The *t*-SNE plot shows malignant and nonmalignant cells were divided into 14 clusters. (b) The *t*-SNE plot displays the 9 cell types of malignant and nonmalignant cells. (c) The *t*-SNE shows the cell types distributed in malignant cell. (d) The *t*-SNE displays the cell types distributed in nonmalignant cell.

The overall flowchart of this study was shown in Figure S1. In accordance with the quality control standard and the standardization of scRNA-seq data, 44516 cells from 50174 osteosarcoma cells were selected for analysis (Figure S2(b) and S2(c)). There was a significant correlation between the number of RNA and UMI counts with Pearson's *R* = 0.88. And the correlation coefficient *R* = −0.23 between the number of RNA features and the content of mitochondrial genes indicated that there was a negative correlation between them, but the correlation degree was low. The correlation coefficient between UMI counts and the content of mitochondrial genes was −0.08, indicating that that their negative correlation was almost negligible (Figure S2(a)). PCA was performed to determine the available dimensions, and the results did not show significant separation between cells in osteosarcoma. Top 50 PCs with significant differences were selected for further analysis (Figure S2(d)). The unsupervised clustering of cells was visualized by t-SNE, showing the distribution of cells in each tissue in different colors (Figure 1(a)). In view of the fact that proliferation is one of the main characteristics of tumor cells, we also labeled cells at different stages of the cell cycle in human osteosarcoma (Figure 1(b)). Furthermore, the distribution of normal cells and cancer cells in osteosarcoma was also annotated (Figure 1(c)). We found that in most samples, the proportion of malignant cells was significantly higher than that of normal cells (Figure 1(d)). Most of the cells from osteosarcoma samples were in G1 phase of the cell cycle. Another part of the cells were in the S phase of the cell cycle, and a small part of the cells were in the G2/*M* phase of the cell cycle (Figure 1(e)). Fourteen clusters were identified for clustering of malignant and nonmalignant cells (Figure 2(a)), which were annotated as nine cell types (B cell, CD8 T cell, endothelial cell, epithelial cell, fibroblast, macrophage, mast cell, osteoblastic tumor cell, and plasma cell) (Figure 2(b)). Osteoblastic tumor cell accounted for the majority of malignant cells and epithelial cells (Figure 2(c)). Significant distribution of endothelial cells, mast cell, macrophage, osteoblastic tumor cell, fibroblast, plasma cell, and B cell can be seen in nonmalignant cells (Figure 2(d)).

### 3.2. Autophagy Was Related to the Malignant Degree of Tumor Cells

**Figure 3:**
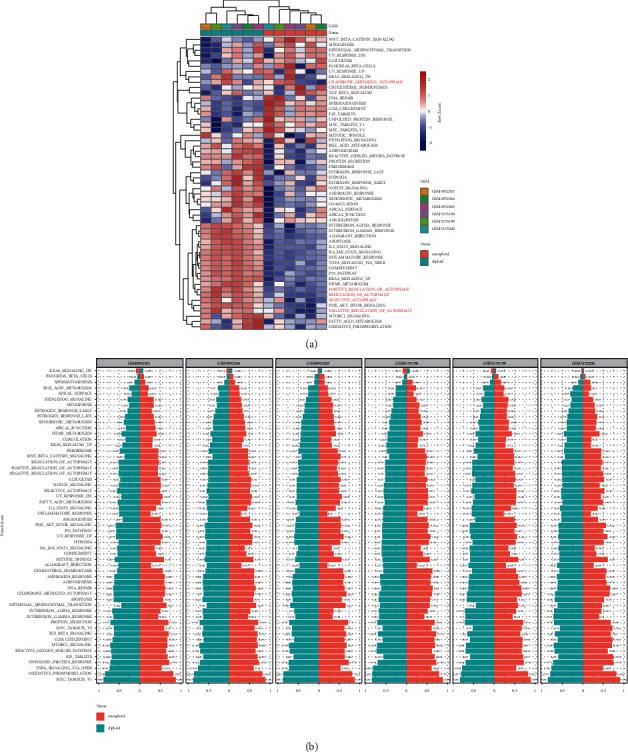
The role of autophagy in normal and malignant cells of osteosarcoma. (a) Difference in activation of biological pathways between normal and malignant cells in osteosarcoma. (b) The enrichment scores of different signal pathways in normal and malignant cells of each osteosarcoma sample.

We analyzed the expression of different tumor regulatory pathways between malignant and nonmalignant cells in osteosarcoma. Different from normal cells, epithelial-mesenchymal transition and DNA repair, G2M checkpoint, E2F targets and MYC targets, and other signaling pathways were highly active in malignant cells. It was worth noting that the activity of a variety of autophagy signals was inhibited, including chaperone-mediated autophagy, positive regulation of autophagy, regulation of autophagy, selective autophagy, and negative regulation of autophagy (Figure 3(a)). These pathways also had different enrichment scores in normal cells and malignant cells (Figure 3(b)). Therefore, autophagy may play an important role in the malignant transformation of cells.

### 3.3. Development and Validation of Autophagy‐Related Risk Signature

**Figure 4:**
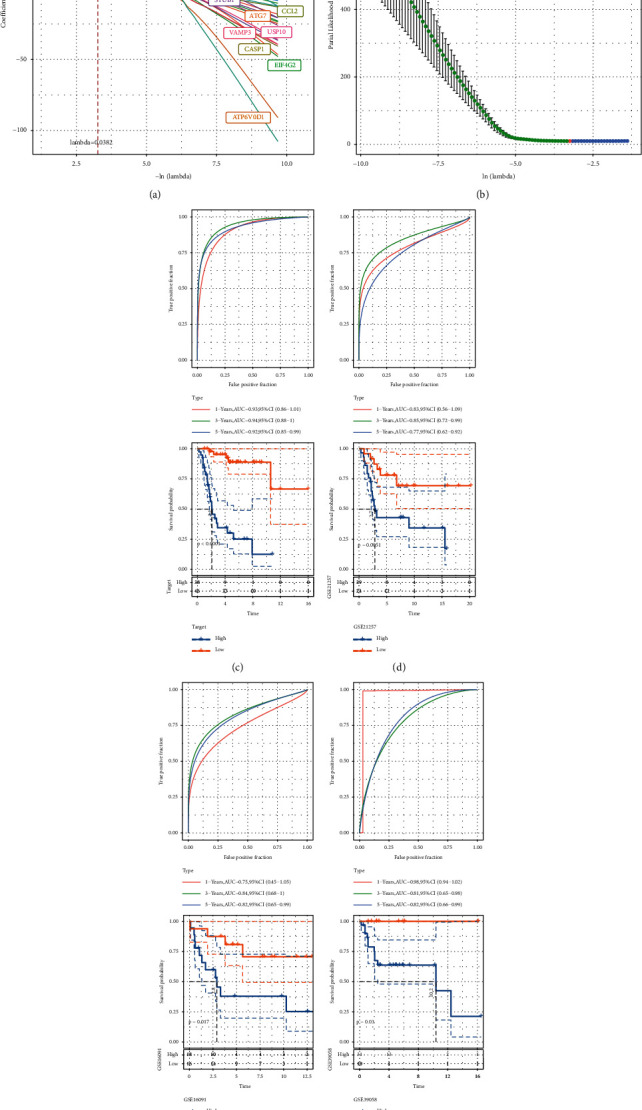
Development and validation of autophagy‐related risk signature. (a) The regression coefficient of each gene was calculated by LASSO regression analysis. (b) The optimal parameter was selected in the LASSO model. (c) The survival curve of samples calculated by risk signature and the ROC curve for evaluating risk signature efficiency in Target dataset. (d–f) Survival analysis and ROC curves for predicting 1 -, 3-, and 5-year survival of osteosarcoma from three datasets from the GEO database.

Based on univariate Cox regression analysis of all acquired ARGs, 39 ARGs related to osteosarcoma survival were screened. LASSO regression analysis was used to calculate the regression coefficient of each gene (Figure 4)(a). The model performed best when 21 genes were included (Figure 4)(b). Further multivariate Cox regression analysis screened 9 genes from 21 ARGs to develop risk signature: risk score = −0.675^*∗*^HUWE1 + 0.585^*∗*^MYC − 1.375^*∗*^EIF4G2 − 0.729^*∗*^USP10 + 0.911^*∗*^KIF25 + 0.595^*∗*^TRIM8−1.218^*∗*^CASP1 − 0.732^*∗*^STUB1 + 1.391^*∗*^CRYBA1. A risk score for each patient was computed using the risk signature to predict prognosis. In the Target dataset, the survival time of patients with low-risk score was significantly longer respective to patients with high-risk score. The 1-year, 3-year, and 5-year classification efficiency scores of risk signature for prognostic prediction were 0.93, 0.94, and 0.92, respectively, far higher than 0.75, indicating that it has strong specificity and sensitivity in prognostic prediction of patients in Target dataset (Figure 4)(c). For the verification set GSE21257, risk score was significantly correlated with the prognosis of the patients. Risk score predicted survival of 1-year AUC = 0.83, 3-year AUC = 0.85, 5-year AUC = 0.77 (Figure 4(d)). For the samples in the GSE16091 dataset and GSE39058 dataset, high-risk score was significantly associated with a worse prognosis. In both cohorts, the AUC of ROC curve of risk signature predicting 1-year, 3-year, and 5-year survival rate of osteosarcoma was all more than 0.75 (Figures 4(e), 4)(f). These data confirmed the satisfactory predictive efficiency of risk signature based on 9 ARG in the prognosis of osteosarcoma.

### 3.4. Prognostic Value of the Risk Signature

**Figure 5:**
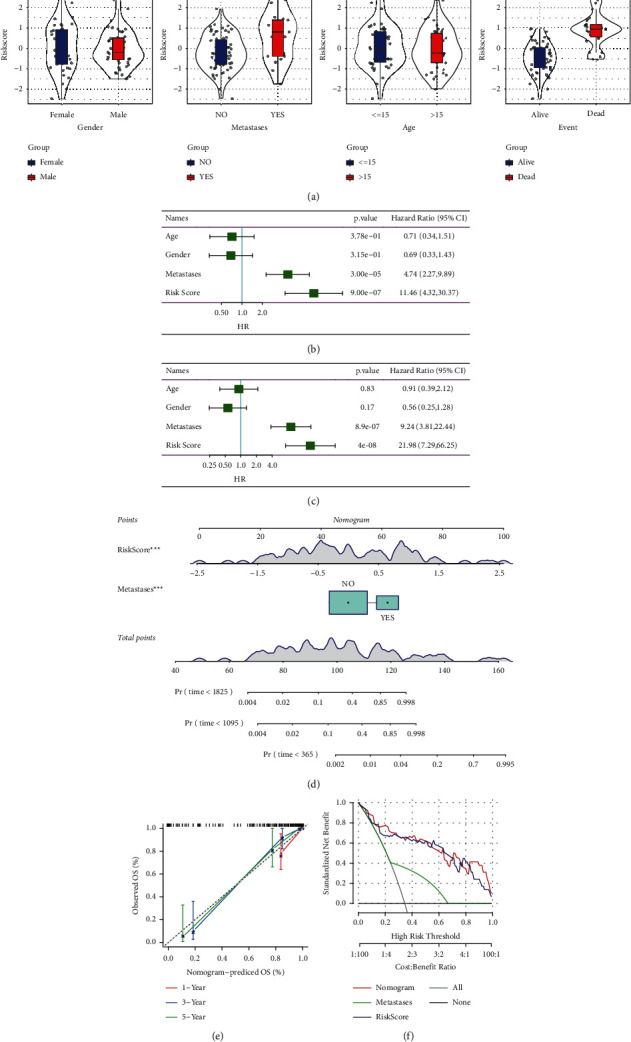
Prognostic value of the risk signature. (a) After stratifying the patients in Target dataset according to gender, newly diagnosed age, metastasis, and survival status, the core risks for the patients in the group were compared. (b–c) Univariate and multivariate Cox regression analysis determined the independent prognostic factors of osteosarcoma in Target dataset. (d) The nomogram constructed based on risk score and metastases predicted the 1 -, 3-, and 5-year survival rates of patients with osteosarcoma. (e) Calibration curves for nomogram. (f) DCA evaluated the net benefit of risk score and metastases and nomogram in predicting the prognosis of osteosarcoma. The asterisks represented the statistical *p* value (ns, no statistical difference, ^*∗*^*p* < 0.05, ^*∗∗*^*p* < 0.01, ^*∗∗∗*^*p* < 0.001, ^*∗∗∗∗*^*p* < 0.0001).

We investigated whether risk signature can distinguish different clinicopathological features. The patients in Target dataset were stratified according to gender, newly diagnosed age, metastasis, and survival status. By comparing the risk score of each group, we found that gender and age had no significant correlation with risk score. The risk score of patients with tumor metastasis was significantly higher than that of patients without metastasis. The risk score of dead patients was also significantly higher than that of survival patients (Figure 5)(a). Univariate and multivariate Cox regression analysis showed that risk score and metastases were independent variables for predicting the prognosis of osteosarcoma (Figures 5(b), 5)(c). To evaluate the prognosis of patients with osteosarcoma more accurately, a nomogram was constructed according to the independent prognostic variables risk score and metastases (Figure 5)(d). The calibration curve of the nomogram showed that the 1-, 3-, and 5-year survival rates predicted by nomogram were in good agreement with the observed survival rates (Figure 5)(e). The net benefit of risk score and metastases and nomogram in predicting the prognosis of osteosarcoma was evaluated by DCA. When the threshold was about 0.11.0, risk score alone or nomogram combined with risk score and metastases showed higher net benefit in the prognosis assessment of patients (Figure 5)(f).

### 3.5. Changes of TME-Related Factors between Different Risk Groups Predicted by Risk Signature

**Figure 6:**
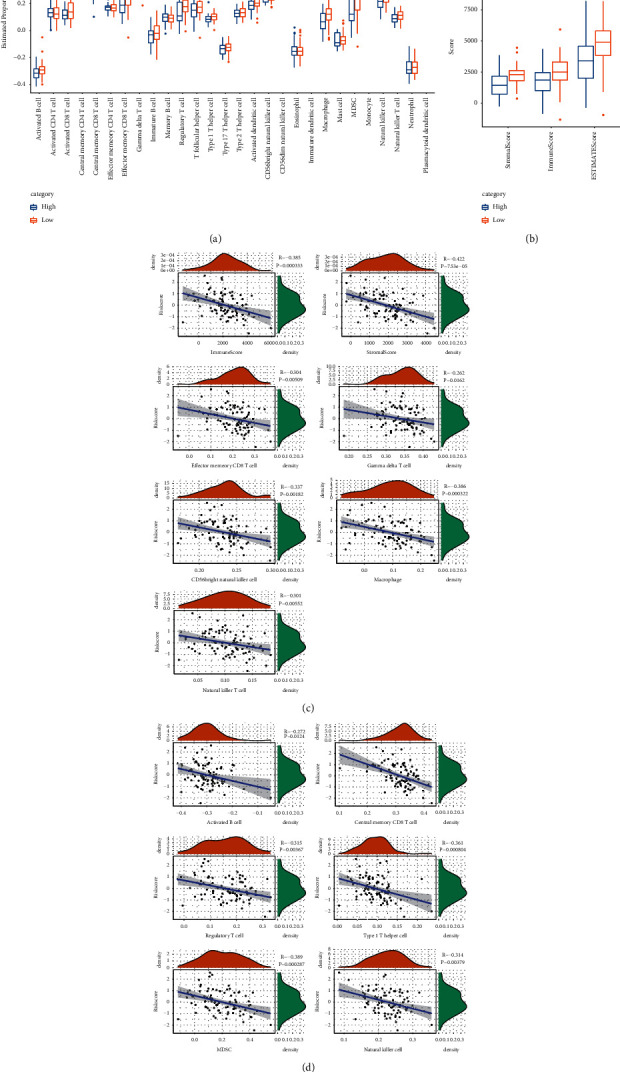
Changes of TME-related factors between different risk groups predicted by risk signature. (a) Risk signature predicts the abundance of infiltrating immune cells in different risk groups. (b) Differences in TME scores between the two risk score groups, including stromal score, immune score, and ESTIMATE score. (c–d) Spearman correlation analysis of risk score and TME-related indicators with significant differences between high- and low-risk score groups and risk score. The asterisks represent the statistical *p* value (ns, no statistical difference, ^*∗*^*p* < 0.05, ^*∗∗*^*p* < 0.01, ^*∗∗∗*^*p* < 0.001).

Considering the importance of infiltrating immune cells in TME, the abundance of infiltrating immune cells in different risk groups predicted by risk signature was evaluated by CIBERSORT. For all infiltrating immune cells that showed a difference in abundance between the high-risk score and low-risk score groups, including activated B cell, central memory CD8 T cell, effector memory CD8 T cell, gamma delta T cell, regulatory T cell, type 1 T helper cell, CD56 bright natural killer cell, macrophage, myeloid-derived suppressor cell (MDSC), natural killer cell, and natural killer T cell, their abundance was significantly higher in the low-risk score group (Figure 6)(a). The TME score (stromal score, immune score, ESTIMATE score) of the two risk score groups was significantly different, and the low-risk score group showed significantly higher TME-related scores relative to the high-risk group (Figure 6)(b). Spearman correlation analysis was conducted on all these TME-related indicators and risk score that showed significant differences between the high- and low-risk score groups, and the results showed that they were significantly negatively correlated with risk score (Figures 6(c), 6(d).

### 3.6. Evaluation of Anti-PD-1/PD-L1 Immunotherapy Based on Risk Signature

**Figure 7:**
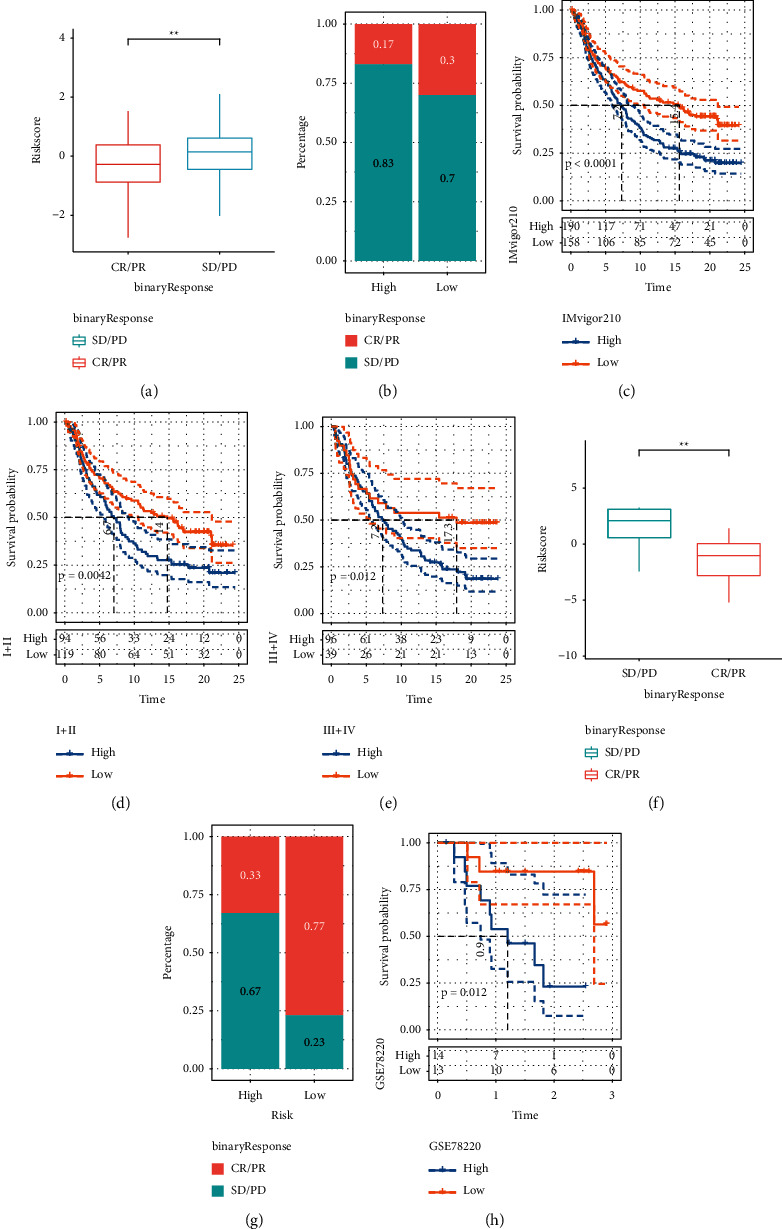
Evaluation of anti-PD-1/PD-L1 immunotherapy based on risk signature. (a) Risk score of patients in the anti-PD-L1 cohort who showed different responses to anti-PD-L1 therapy. (b) The response rate of high-risk score group and low-risk score group to anti-PD-L1 therapy in the anti-PD-L1 cohort. (c) Survival analysis of patients with low-risk score and high-risk score in the IMvigor210 cohort. (d) The survival curve of patients with low-risk score and high-risk score in the IMvigor210 cohort with stage I-II. (e) The Kaplan-Meier curve of stage III-IV patients with different risk score in IMvigor210 cohort. (f) Risk score differences between different ICB treatment groups in the anti-PD1 cohort. (g) The percentage of patients who responded to PD-1 blocking immunotherapy in different risk groups. (h) The Kaplan-Meier curve showed the prognosis of patients in the anti-PD1 cohort classified as high-risk and low-risk groups. The asterisks represent the statistical *p* value (ns, no statistical difference, ^*∗*^*p* < 0.05, ^*∗∗*^*p* < 0.01).

We studied whether risk signature can predict patients' response to ICB treatment based on two immunotherapy cohorts. In the anti-PD-L1 cohort, 348 patients showed different responses to anti-PD-L1 treatment, including complete response (CR), partial response (PR), stable disease (SD), and progressive disease (PD). Risk score in patients with SD/PD was significantly higher than that in patients with CR/PR (Figure 7(a)). The response rate of high-risk score group and low-risk score group to anti-PD-L1 treatment was further determined. The results showed that the response rate to ICB therapy was 30% in the low-risk score group and 17% in the high-risk score group (Figure 7(b)). And the patients with low-risk score in the IMvigor210 cohort showed significant survival advantages compared with the patients with high-risk score (Figure 7(c)). For both early and advanced patients in this cohort, high-risk score was always associated with shorter survival (Figures 7(d), 7(e)). In the anti-PD1 cohort, risk score was significantly upregulated in patients with SD/PD treated with anti-PD-1 compared with patients with CR/PR treated with anti-PD1 (Figure 7(f)). The response rate of the low-risk score group to immunotherapy in this cohort was 77%, which was significantly higher than that of the high-risk score group (Figure 7(g)). In addition, the high-risk score in this cohort caused a poor prognosis (Figure 7(h)). An analysis of these results between the two risk groups of autophagy-related risk signature classification showed that patients with low-risk score had a higher potential to respond to immunotherapy.

## 4. Discussion

For a long time, the prognosis of patients with metastatic osteosarcoma has not been satisfactory [16]. Advances in biological understanding, the development of robust preclinical models, the feasibility of rapid clinical testing and novel treatment concepts are beneficial to improve the prognosis of patients with osteosarcoma [8]. Autophagy is a biological behavior related to osteosarcoma metastasis and has a complex relationship with TME and drug resistance [17, 18]. Based on emerging bioinformatics analysis of technologies and methods, it is expected to expand our understanding of the biological effects of autophagy and its relationship with TME.

In this study, we identified autophagy regulatory genes related to the prognosis of osteosarcoma and constructed risk signature based on 9 prognosis-related autophagy regulatory genes. Its accuracy and practicability were verified in 3 datasets. Our results showed that the classifier could effectively distinguish osteosarcoma patients with different survival outcomes and showed high specificity and sensitivity. Reviewing the nine ARGs in risk signature, HUWE1 is a ubiquitin (Ub) E3 ligase, which acts as a terminating enzyme during protein ubiquitination [19]. According to currently available studies, HUWE1 plays a key role in regulating autophagy and other biological functions in many cancers, including DNA damage response, transcription, autophagy, apoptosis, and metabolism [20]. Transcription factor MYC plays a central role in cancer by inducing autophagy [21]. EIF4G2 is a kind of eukaryotic translation initiation factor that indirectly participates in the regulation of autophagy in cancer [22]. USP10 can enhance autophagy under stress [23]. Inhibition of KIF25 can kill cervical cancer and osteosarcoma cells [24]. It is reported that TRIM8 participates in many cellular responses, plays a central role in immune response, and coordinates various basic biological processes, including autophagy [25]. STUB1 is a chaperone-dependent E3 ubiquitin ligase, which participates in the regulation of autophagy and lysosome function by regulating the activity of TFEB [26]. CRYBA1/*β* A3/A1-crystallin locates in lysosomes and controls phagocytosis and autophagy by regulating internal lysosomal acidification and V-ATP enzyme [27]. The comprehensive effect of autophagy regulated by these genes in osteosarcoma was reported for the first time in this study.

Malignant osteosarcoma cells form a complex mixture with other normal cells and some chemical factors (hypoxia, acidosis). This complex mixture of special TME is a perfect place for osteosarcoma to develop and metastasize [28]. Therefore, these aspects should not be ignored when discussing the regulation of osteosarcoma. Here, in the first part of the study, we distinguished the distribution of normal and malignant cells in osteosarcoma tissues by scRNA-seq analysis. After the establishment of risk signature, the predictive value of the model on the abundance of infiltrating immune cells and TME score in TME was analyzed. Activated B cell, central memory CD8 T cell, effector memory CD8 T cell, gamma delta T cell, regulatory T cell, type 1 T helper cell, CD56 bright natural killer cell, macrophage, MDSC, natural killer cell, and natural killer T cell had a high proportion in the low-risk group based on risk signature, and in the low-risk group, the immune score and stromal score in TME were also significantly higher than those in the high-risk group. Autophagy has been reported in previous studies to directly regulate the activity of T cells and natural killer cells [29]. And autophagy in T cell subsets plays an active role in antitumor immune response [30]. The risk score composed of ARGs was negatively correlated with the abundance of 6 kinds of T cells and natural killer cell, suggesting that 9 ARGs were the regulatory factors of autophagy to control the activity of the above immune cells, and the antitumor immune effect was stronger in the low-risk group.

The key role of autophagy in TME is related to the efficacy of antitumor immunotherapy [31]. We investigated whether risk signature dominated by ARGs helps guide ICB therapeutic interventions. In the two immunotherapy cohorts studied, the response rate of samples with low-risk score to anti-PD-L1 or anti-PD1 was always significantly higher than that of patients with high-risk score. Therefore, low-risk groups are more likely to benefit from ICB.

In summary, we have identified a new ARGs-driven risk signature, which can not only help to evaluate the prognosis of osteosarcoma, but also can be used as a useful tool to distinguish patients' different clinical features and TME. More importantly, risk signature can help predict patients' responses to immunotherapy.

## Data Availability

The datasets generated and/or analyzed during the current study are available in the [GSE162454] repository (https://www.ncbi.nlm.nih.gov/geo/query/acc.cgi?acc=GSE162454), [GSE21257] repository (https://www.ncbi.nlm.nih.gov/geo/query/acc.cgi?acc=GSE21257), [GSE39058] repository (https://www.ncbi.nlm.nih.gov/geo/query/acc.cgi?acc=GSE39058), and [GSE16091] repository (https://www.ncbi.nlm.nih.gov/geo/query/acc.cgi?acc=GSE16091).

## Supplementary Materials

Supplementary Materials Figure S1. The overall flowchart of this study. Figure S2. ScRNA-seq analysis of 6 osteosarcoma samples. A: the correlation between mitochondrial gene and the number of UMI/mRNA, and the relationship between the number of UMI and mRNA. B, C: quality control, including the number of unique genes and total molecules, and the percentage of reads that map to the mitochondrial genome. D: the PCA based on scRNA-seq data confirms top 50 PCs. Table S1. The name of 531 autophagy‐related genes.
