# Supplementary material for: Bioinformatics Analysis Reveals an Association between Autophagy, Prognosis, Tumor Microenvironment, and Immunotherapy in Osteosarcoma
Source: J Oncol. 2022 Jul 14;2022:4220331. doi: 10.1155/2022/4220331 (PMC9303156; doi:10.1155/2022/4220331)
Supplement: Supplementary Materials — Figure S1. The overall flowchart of this study. Figure S2. ScRNA-seq analysis of 6 osteosarcoma samples. A: the correlation between mitochondrial gene and the number of UMI/mRNA, and the relationship between the number of UMI and mRNA. B, C: quality control, including the number of unique genes and total molecules, and the percentage of reads that map to the mitochondrial genome. D: the PCA based on scRNA-seq data confirms top 50 PCs. Table S1. The name of 531 autophagy‐related genes. [file 4220331.f1.zip › Table S1.pdf]

Table S1 Autophagy-related genes
AMBRA1
APOL1
ARNT
ARSA
ARSB
ATF4
ATF6
ATG10
ATG12
ATG16L1
ATG16L2
ATG2A
ATG2B
ATG3
ATG4A
ATG4B
ATG4C
ATG4D
ATG5
ATG7
ATG9A
ATG9B
ATIC
BAG1
BAG3
BAK1
BAX
BCL2
BCL2L1
BECN1
BID
BIRC5
BIRC6
BNIP1
BNIP3
BNIP3L
C12orf44
C17orf88
CALCOCO2
CAMKK2
CANX
CAPN1
CAPN10
CAPN2
CAPNS1
CASP1
CASP3
CASP4
CASP8
CCL2
CCR2
CD46
CDKN1A
CDKN1B
CDKN2A
CFLAR
CHMP2B

CHMP4B
CLN3
CTSB
CTSD
CTSL1
CX3CL1
CXCR4
DAPK1
DAPK2
DDIT3
DIRAS3
DLC1
DNAJB1
DNAJB9
DRAM1
EDEM1
EEF2
EEF2K
EGFR
EIF2AK2
EIF2AK3
EIF2S1
EIF4EBP1
EIF4G1
ERBB2
ERN1
ERO1L
FADD
FAM48A
FAS
FKBP1A
FKBP1B
FOS
FOXO1
FOXO3
GAA
GABARAP
GABARAPL1
GABARAPL2
GAPDH
GNAI3
GNB2L1
GOPC
GRID1
GRID2
HDAC1
HDAC6
HGS
HIF1A
HSP90AB1
HSPA5
HSPA8
HSPB8
IFNG
IKBKB
IKBKE
IL24
IRGM

ITGA3
ITGA6
ITGB1
ITGB4
ITPR1
KIAA0226
KIAA0652
KIAA0831
KIF5B
KLHL24
LAMP1
LAMP2
MAP1LC3A
MAP1LC3B
MAP1LC3C
MAP2K7
MAPK1
MAPK3
MAPK8
MAPK8IP1
MAPK9
MBTPS2
MLST8
MTMR14
MTOR
MYC
NAF1
NAMPT
NBR1
NCKAP1
NFE2L2
NFKB1
NKX2-3
NLRC4
NPC1
NRG1
NRG2
NRG3
P4HB
PARK2
PARP1
PEA15
PELP1
PEX14
PEX3
PIK3C3
PIK3R4
PINK1
PPP1R15A
PRKAB1
PRKAR1A
PRKCD
PRKCQ
PTEN
PTK6
RAB11A
RAB1A
RAB24

RAB33B
RAB5A
RAB7A
RAC1
RAF1
RB1
RB1CC1
RELA
RGS19
RHEB
RPS6KB1
RPTOR
SAR1A
SERPINA1
SESN2
SH3GLB1
SIRT1
SIRT2
SPHK1
SPNS1
SQSTM1
ST13
STK11
TBK1
TM9SF1
TMEM49
TMEM74
TNFSF10
TP53
TP53INP2
TP63
TP73
TSC1
TSC2
TUSC1
ULK1
ULK2
ULK3
USP10
UVRAG
VAMP3
VAMP7
VEGFA
WDFY3
WDR45
WDR45L
WIPI1
WIPI2
ZFYVE1
ATP13A2
CLU
CTSA
EEF1A1
EEF1A2
GFAP
HSP90AA1
PLK3
SNCA

SNRNP70
STUB1
SYNPO2
ADRA1A
AKT1
BMF
CHMP4A
CLEC16A
CPTP
DAP
DAPL1
EHMT2
EIF4E
EIF4G2
EIF4G3
FEZ1
FEZ2
FOXK1
FOXK2
GATA4
GOLGA2
HERC1
HGF
HMOX1
HTR2B
IL10
IL10RA
KDM4A
KIF25
KLHL22
LEP
LEPR
LRRK2
LZTS1
MAGEA3
MAGEA6
MCL1
MET
MIR199A1
MIRLET7B
MT3
MTM1
MTMR8
MTMR9
NRBP2
NUPR1
PHF23
PIK3CA
POLDIP2
PTPN22
QSOX1
RASIP1
RNF41
RNF5
RRAGA
RUBCN
SCFD1
SEC22B

SMCR8
SMG1
STAT3
TAB2
TAB3
TBC1D14
TIGAR
TLK2
TMEM39A
TREM2
TSPO
UBQLN4
USP30
USP36
WASHC1
WDR6
ZKSCAN3
ADRB2
BAD
BCL2L11
C9orf72
CERS1
DCN
DEPDC5
DHRSX
ELAPOR1
ENDOG
EPM2A
FBXO7
FLCN
FYCO1
GPSM1
GSK3A
GSK3B
HMGB1
HTT
IKBKG
IL4
KAT5
KDR
LACRT
LARP1
LRSAM1
MAP3K7
MEFV
MID2
MOAP1
MTDH
NOD1
NOD2
NPRL2
NPRL3
OPTN
ORMDL3
PAFAH1B2
PARK7
PIK3C2A
PIK3CB

PIM2
PIP4K2A
PIP4K2B
PIP4K2C
PLEKHF1
PLK2
PRKAA1
PRKAA2
PRKD1
PRKN
RAB3GAP1
RAB3GAP2
RALB
RIPK2
RNF152
RNF31
ROCK1
RUFY4
SCOC
SESN1
SESN3
SH3BP4
SLC25A4
SLC25A5
SNX18
SNX30
SNX4
SNX7
SPTLC1
SPTLC2
STING1
SUPT5H
SVIP
TFEB
TICAM1
TMEM59
TP53INP1
TPCN1
TRIM13
TRIM14
TRIM21
TRIM22
TRIM27
TRIM32
TRIM34
TRIM38
TRIM5
TRIM6
TRIM65
TRIM68
TRIM8
TRIML1
TRIML2
UFL1
VDAC1
VPS13D
WAC
ZC3H12A

ABL1
ABL2
ACER2
ATG14
ATM
ATP6V0A1
ATP6V0A2
ATP6V0B
ATP6V0C
ATP6V0D1
ATP6V0D2
ATP6V0E1
ATP6V0E2
ATP6V1A
ATP6V1B1
ATP6V1B2
ATP6V1C1
ATP6V1C2
ATP6V1D
ATP6V1E1
ATP6V1E2
ATP6V1G1
ATP6V1G2
ATP6V1H
BOK
CDK5
CDK5R1
CISD1
CISD2
CRYBA1
CSNK2A2
CTTN
DAPK3
DCAF12
DEPP1
DNM1L
DRAM2
EP300
ERCC4
EXOC1
EXOC4
EXOC7
EXOC8
FBXL2
FBXW7
FZD5
GBA
GPR137
GPR137B
HAX1
HSPB1
HTRA2
IFI16
KAT8
KEAP1
LAMP3
MAPK15
MAPT

MFSD8
MTCL1
MTMR3
MTMR4
NEDD4
NLRP6
OSBPL7
PARL
PIK3R2
PRKACA
PSAP
PYCARD
RAB39B
RAB8A
RMC1
RRAGB
RRAGC
RRAGD
SNX32
SNX5
SNX6
SOGA1
SOGA3
SREBF1
SREBF2
TBC1D25
TECPR1
TPCN2
TRIB3
UBQLN1
UBQLN2
UCHL1
USP13
USP33
VPS13C
VPS26A
VPS26B
VPS29
VPS35
WDR24
WDR41
ZMPSTE24
ACBD5
ARFIP2
ATG13
AUP1
C5orf51
CDC37
DDRGK1
HUWE1
KLHL3
LGALS8
LRBA
MFN2
PHB2
PJVK
RETREG1
RETREG3

RNF213
STBD1
TAFAZZIN
TEX264
TOMM7
UBA5
UFC1
UFM1
WDR81
