# Supplementary figures and images for: Bioinformatics Analysis Reveals an Association between Autophagy, Prognosis, Tumor Microenvironment, and Immunotherapy in Osteosarcoma
Source: J Oncol. 2022 Jul 14;2022:4220331. doi: 10.1155/2022/4220331 (PMC9303156; doi:10.1155/2022/4220331)

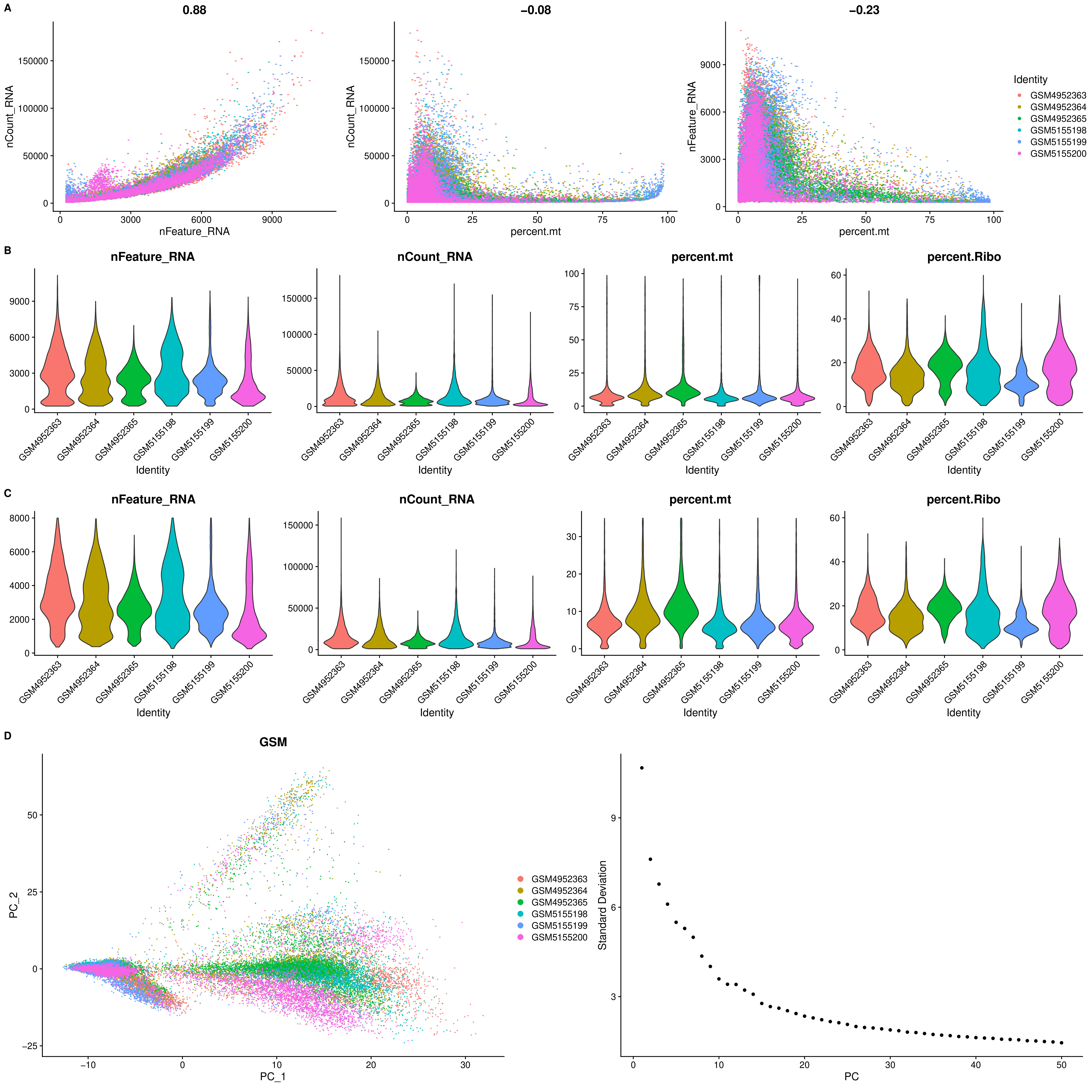

Supplement: Supplementary Materials — Figure S1. The overall flowchart of this study. Figure S2. ScRNA-seq analysis of 6 osteosarcoma samples. A: the correlation between mitochondrial gene and the number of UMI/mRNA, and the relationship between the number of UMI and mRNA. B, C: quality control, including the number of unique genes and total molecules, and the percentage of reads that map to the mitochondrial genome. D: the PCA based on scRNA-seq data confirms top 50 PCs. Table S1. The name of 531 autophagy‐related genes. [file 4220331.f1.zip › Figure S1.jpg]
